# Pioneering Periapical Healing: The Novel Synergy of Mineral Trioxide Aggregate and Injectable Platelet-Rich Fibrin

**DOI:** 10.7759/cureus.46341

**Published:** 2023-10-01

**Authors:** Paridhi Agrawal, Pradnya Nikhade, Aditya Patel, Jay Bhopatkar, Tejas Suryawanshi

**Affiliations:** 1 Department of Conservative Dentistry and Endodontics, Sharad Pawar Dental College and Hospital, Datta Meghe Institute of Higher Education and Research, Wardha, IND

**Keywords:** radicular cyst, non-surgical management, mineral trioxide aggregate, injectable platelet-rich fibrin, periapical lesion

## Abstract

This case report presents a novel non-surgical approach for managing a substantial periapical lesion associated with tooth 12 using a combination of injectable platelet-rich fibrin (i-PRF) and mineral trioxide aggregate (MTA). A 28-year-old male patient presented with pus discharge and intermittent swelling following a history of dental trauma. Clinical and radiographic assessments confirmed a large periapical cyst associated with tooth 12. The treatment involved root canal therapy with calcium hydroxide medication, leading to symptom relief. Subsequently, i-PRF combined with MTA was used as a regenerative material for periapical healing. Follow-up examinations at three, six, and nine months showed complete resolution of symptoms and radiographic evidence of lesion healing. This innovative technique demonstrates the potential of i-PRF and MTA synergy in the non-surgical management of periapical lesions, avoiding the risks associated with surgical interventions and promoting effective tissue healing.

## Introduction

Traumatic injuries typically disrupt the blood supply to the dental pulp, leading to pulp tissue death and creating oxygen-deprived conditions that favor the growth of opportunistic microorganisms. Consequently, this can result in the formation of periapical lesions [1]. Periapical lesions generally arise as a response to the intrusion of microorganisms and their by-products into the root canal system, with their growth being facilitated through various processes such as the accumulation of fluid due to osmosis, the proliferation of epithelial cells, and molecular mechanisms [2]. Therefore, if the inflammatory exudates within the lesion are effectively removed to reduce hydrostatic pressure, and if the underlying microbiological cause is addressed through nonsurgical root canal treatment, these lesions may shrink and regress by apoptosis [3].

The ultimate objective of endodontic treatment is to restore the affected tooth to a healthy and functional state without undergoing surgical procedures [4]. Until recently, the conventional approach to managing endodontic periapical lesions, especially when they were large, involved surgical intervention. However, recent advances in our understanding of these lesions' origin, pathological characteristics, and clinical behavior, coupled with successful treatments in various clinical trials, have shifted the preference toward nonsurgical methods [5]. All inflammatory periapical lesions should be addressed using conservative non-surgical methods, such as orthograde root canal therapy because it is less invasive, has more patient acceptability, and causes less psychological distress to the patients [6]. Surgical intervention is only recommended when non-surgical approaches prove ineffective [7]. Furthermore, surgical procedures come with several drawbacks such as fistula formation, healing by scar tissue formation, and sometimes swelling that limit their use in the treatment of periapical lesions [8]. It is worth noting that endodontic treatment for teeth with periapical lesions has been reported to achieve a success rate of 85% [9]. Additionally, there have been reports of a 94.4% incidence of complete and partial healing of periapical lesions following non-surgical endodontic therapy [10]. This case report describes a novel technique for the non-surgical management of a large periapical lesion using a combination of injectable platelet-rich fibrin (i-PRF) and mineral trioxide aggregate (MTA).

## Case presentation

**Figure 1:**
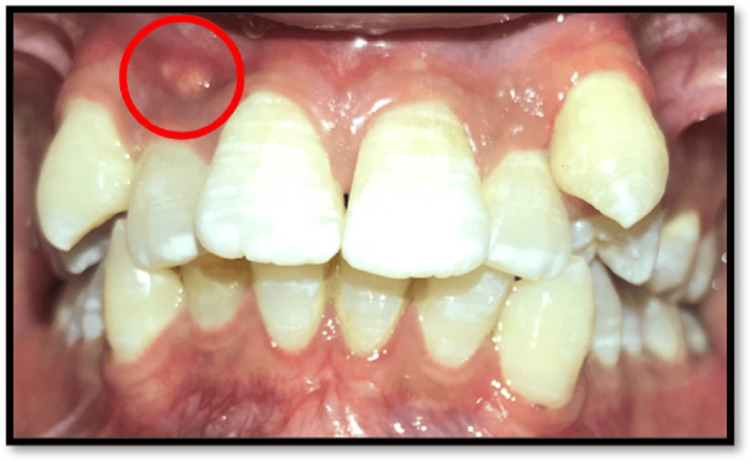
Pre-operative intraoral picture

A 28-year-old male patient presented to the department with a chief complaint of pus discharge from the upper front region of the jaw for one week. The patient had started noticing this pus discharge and accompanying swelling from the upper front region of the jaw for one week, which appeared intermittently, sometimes causing mild discomfort. The patient gave a history of trauma (due to a self-fall) in the same region approximately 10 years ago resulting in mild pain in the upper front region, which was effectively relieved with medication. There was no history of heat or balm application. The past medical and dental history was not significant. On extra-oral examination, no gross asymmetry was detected. On intra-oral examination, a sinus opening was seen associated with tooth 12 (Figure 1).

**Figure 2:**
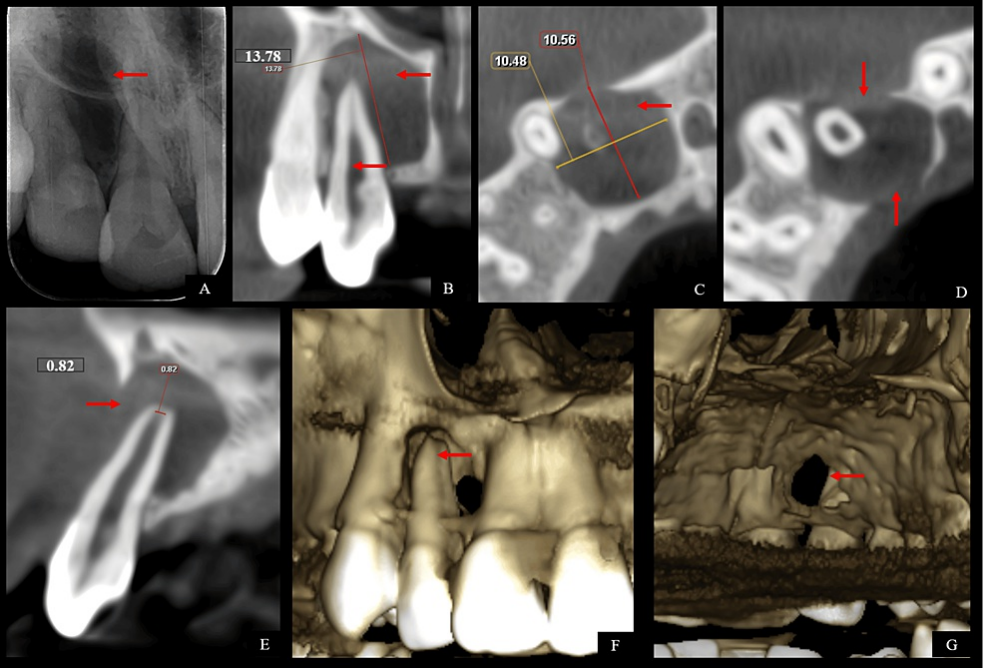
Pre-operative radiographic images (A) Intra-oral periapical radiograph with 12 showing a large periapical radiolucent shadow, (B) coronal cone-beam computed tomographic view showing wide canal and a radiolucent shadow with well-corticated borders measuring 13.78 mm superior-inferiorly and resorbed root apex with 12, (C) axial view showing the lesion measuring 10.48 mm mesiodistally and 10.56 mm buccopalatally, (D) axial view showing perforation of the labial and lingual cortical plate, (E) sagittal view of 12 showing root resorption with an apical diameter of 0.82 mm and loss of buccal cortical plate, (F) bony view showing a tunnel defect with 12, and (G) bony view showing a tunnel defect with 12.

Crowding was evident in the upper and lower jaws. Electric and thermal pulp vitality testing showed a negative response for tooth 12 with a normal response for teeth 11, 13 suggestive of non-vital tooth with 12. All teeth did not elicit tenderness during percussion testing. Subsequently, an intra-oral periapical (IOPA) radiograph was taken which revealed a large periapical radiolucent shadow associated with 12 (Figure 2A). Thereafter, a cone-beam computed tomography (CBCT) was performed with 12 regions. The coronal CBCT view showed a wide canal and a radiolucent shadow with well-corticated borders measuring 13.78 mm superior-inferiorly and a resorbed root apex with 12 (Figure 2B). The axial view showed perforation of the labial and lingual cortical plate and the lesion measuring 10.48 mm mesiodistally and 10.56 mm buccopalatally (Figures 2C, 2D). The sagittal view of 12 showed root resorption with an apical diameter of 0.82 mm and loss of buccal cortical plate (Figure 2E). The bony view showed a tunnel defect with 12 (Figures 2F, 2G).

**Figure 3:**
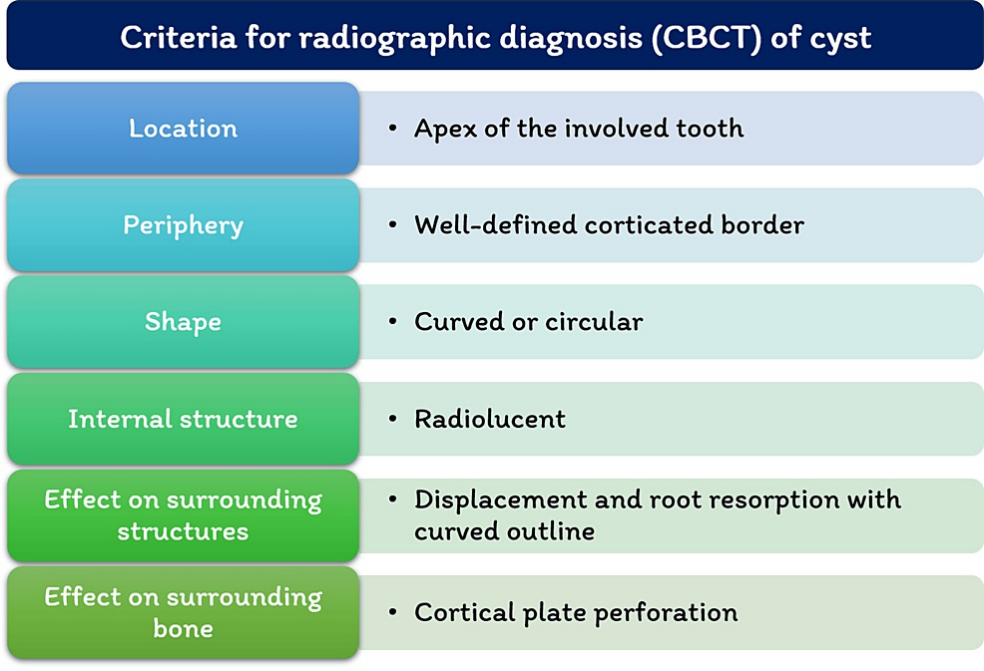
Criteria for radiographic diagnosis of radicular cyst CBCT- Cone-beam computed tomography Image Credits: Paridhi Agrawal

Following the radiographic criteria for the diagnosis of periapical cysts (Figure 3) [11], the findings were suggestive of a periapical cyst of size 10.48 mm x 10.56 mm x 13.78 mm with 12.

Considering the history, clinical examination, and clinical and radiographic investigation, a diagnosis of an infected periapical cyst with 12 was made. A treatment plan was formulated and explained to the patient, and the procedure commenced following the patient's informed consent.

**Figure 4:**
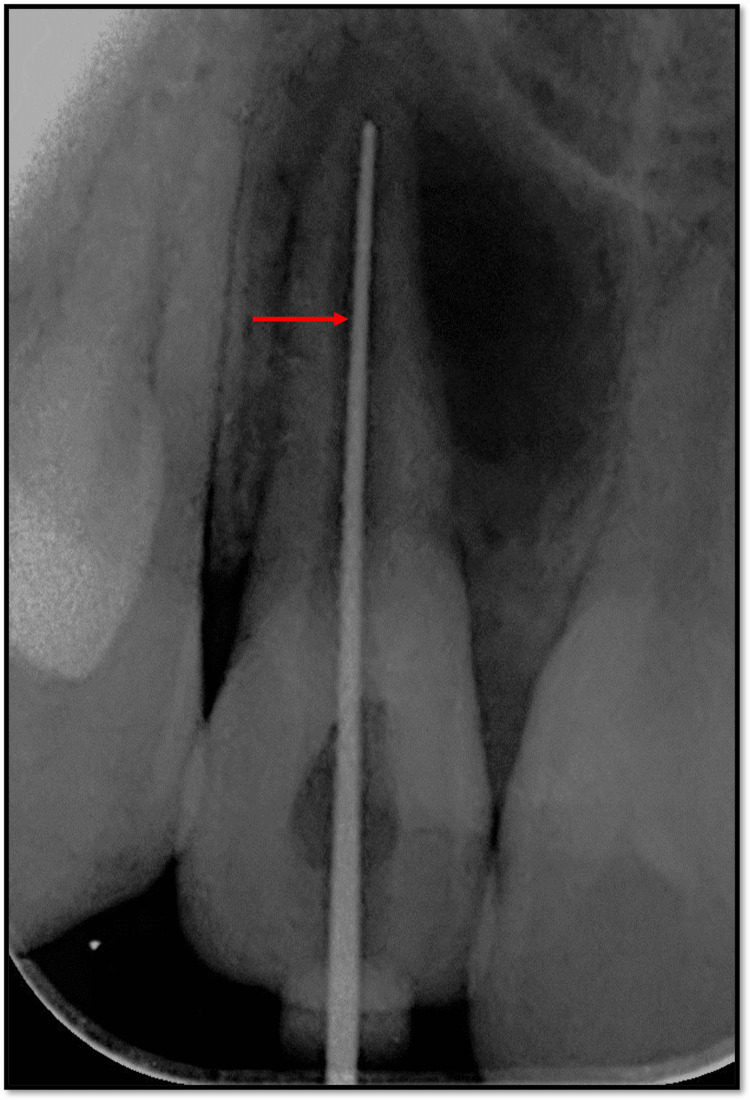
Radiographic confirmation of working length with 12

Under rubber dam isolation, access opening was performed with 12. Thereafter, patency filling was done and working length was determined using an apex locator (Root ZX2, J.W. Morita, Japan) which was then confirmed radiographically. The working length of 12 was found to be 19.5 mm (Figure 4).

**Figure 5:**
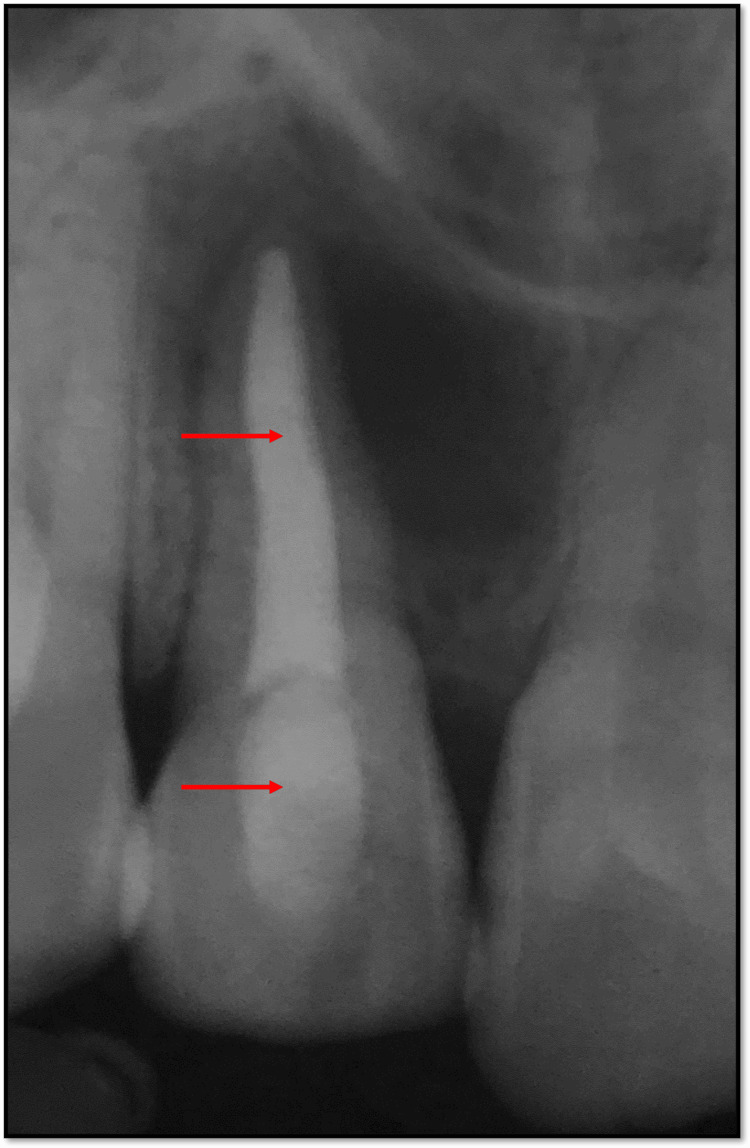
Radiograph showing mineral trioxide aggregate obturation and composite resin post-endodontic restoration with 12

Considering the apical diameter of 0.82 mm, tooth 12 was lightly cleaned and shaped in circumferential strokes using a size 70 hand K-file (Mani, Japan). Over instrumentation two to three times 1mm beyond the apex was performed [12]. Ultrasonically activated irrigation using 0.9% normal saline, 2.5% sodium hypochlorite, and 2% chlorhexidine was performed followed by the placement of calcium hydroxide intracanal medicament dressing for 1 week with tooth 12. In the subsequent visit, complete healing of the sinus tract was seen. Following this, two additional applications of calcium hydroxide intracanal medication spaced at an interval of 1 week were done. In the fourth visit, the tooth was completely asymptomatic, there was no drainage from the canal, so it was decided to perform obturation with 12. A final irrigation with 17% ethylenediamine tetraacetic acid (EDTA) and 0.9% normal saline was carried out with 12 and the canals were dried using absorbent paper points. Thereafter, 1.5 grams of MTA (ProRoot MTA, Dentsply Sirona, USA) was taken and placed in a 2.5mL syringe with 24 gauze needle. Miron’s protocol [13] for the preparation of i-PRF was used. 9mL of the patient's blood was withdrawn and collected in plastic test tubes. It was then centrifuged at 700 rpm for three minutes. The obtained i-PRF in light yellow color was then extracted in the syringe containing MTA, lightly mixed, and placed in the periapical area of tooth 12. Subsequently, the canal was obturated using MTA mixed with i-PRF [14] in a wet sand consistency. The post-endodontic restoration was carried out using composite resin (3M Z250 XT, 3M, USA), and an IOPA radiograph was taken (Figure 5).

**Figure 6:**
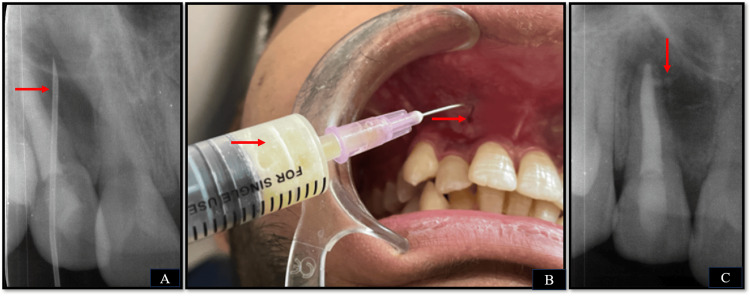
Extra-oral administration of combination of injectable platelet-rich fibrin and mineral trioxide aggregate (A) Radiographic confirmation of needle placement in the cystic cavity, (B) re-administration of the mixture in the periapical area through the buccal mucosa, and (C) radiograph confirming the placement of the mixture in the periapical area.

As i-PRF is recognized for its sustained release of growth factors over a period of approximately 10 days [14], the patient was recalled after 10 days. During this appointment, a fresh mixture of MTA and i-PRF was prepared using the same established protocol as previously mentioned. Subsequently, the mixture was slowly re-administered in the periapical area through the buccal mucosa, ensuring precise needle placement within the cystic cavity adjacent to tooth 12, as confirmed through both palpation and radiographic assessment (Figures 6A-6C).

**Figure 7:**
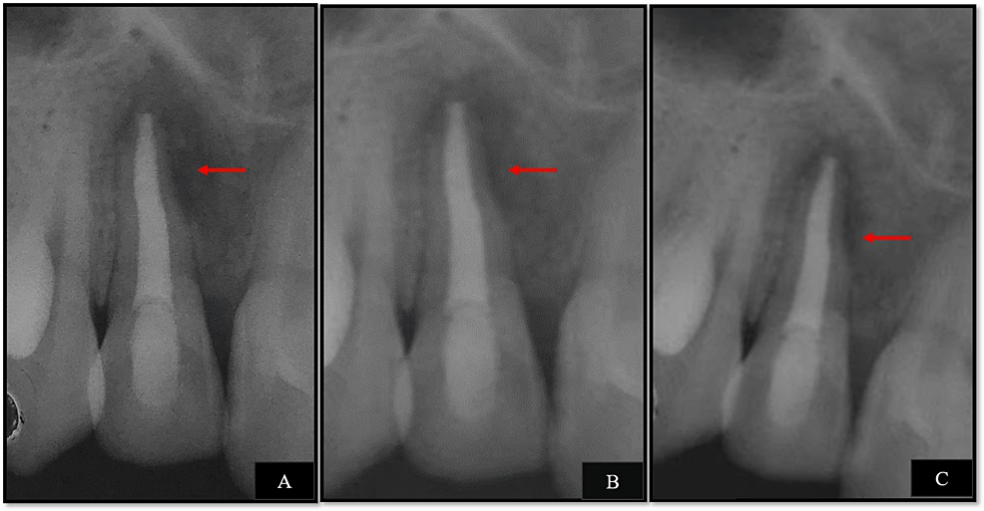
Follow-up radiographs (A) Three months follow-up, (B) six months follow-up, and (C) nine months follow-up

The patient was then recalled for follow-ups after three, six, and nine months during which the clinical and radiographic examination revealed that the patient was completely asymptomatic, and the periapical lesion showed definite signs of healing (Figures 7A-7C).

## Discussion

**Figure 8:**
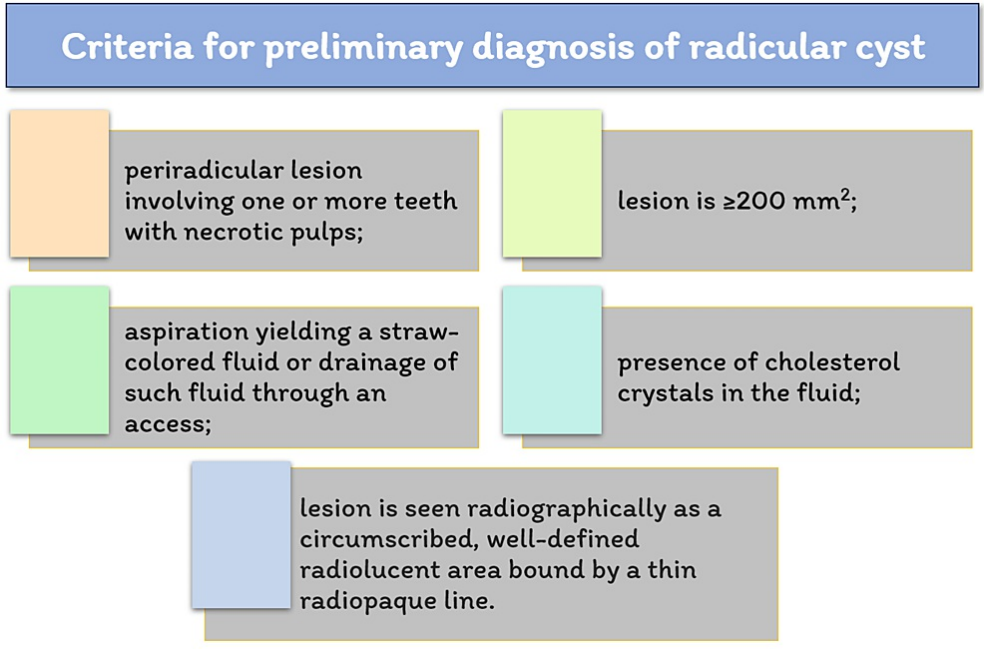
Criteria for preliminary diagnosis of radicular cyst Image Credits: Paridhi Agrawal

Periapical lesions are typically categorized as radicular cysts, dental granulomas, or abscesses [15]. Among these periapical lesions, the prevalence of cysts can range from 6% to 55% [16]. Similarly, the occurrence of granulomas varies between 9.3% and 87.1%, while abscesses are found in approximately 28.7% to 70.07% of cases [17]. It is important to note that based on clinical observations, larger-sized lesions are more likely to be radicular cysts. However, some of these sizable lesions may display characteristics resembling granulomas [18]. The conclusive and definite diagnosis of a cyst can be made only through histopathological findings. As shown in Figure 8, a primary clinical diagnosis of a radicular cyst can be approximately made based on certain facts [18,19].

Additionally, studies have indicated that the occurrence of cysts can be as high as 60%-67% in lesions with a diameter ranging from 10 to 20 mm [20,21]. When taking into account the volume of the lesion, there is an 80% likelihood of it being a cyst when the volume exceeds 247 mm³. In cases where there is root displacement and the volume is less than 247 mm³, there is still a 60% probability of it being a cyst [22].

Nonsurgical management of periapical lesions is the preferred approach when compared to surgical methods and should be contemplated in all cases [23]. This preference is rooted in the potential risks associated with surgical procedures, such as the risk of harming adjacent vital teeth and damage to anatomical structures in the proximity of the lesion. Additionally, the discomfort and pain often associated with surgical interventions can be avoided through nonsurgical means. Patient comfort and their reluctance towards surgical procedures, along with considerations like age and medical conditions that may limit surgical options, further support the choice of a nonsurgical approach. Surgical interventions should only be considered as a last resort when conventional methods have proven ineffective [23].

**Figure 9:**
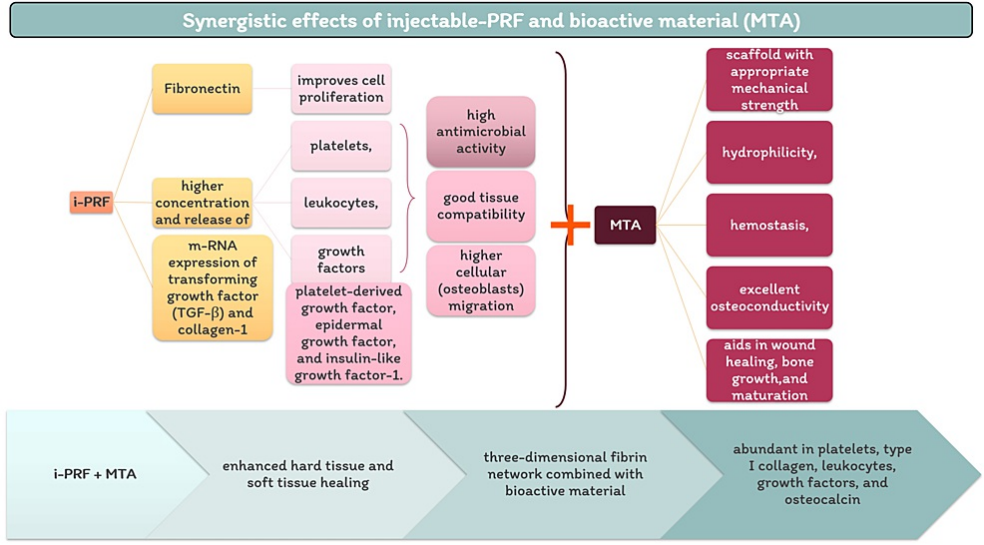
Synergistic effects of injectable platelet-rich fibrin and mineral trioxide aggregate PRF- platelet-rich fibrin, i-PRF- injectable platelet-rich fibrin, MTA- mineral trioxide aggregate Image Credits: Paridhi Agrawal

Calcium hydroxide is a widely utilized intra-canal medication due to its notable high alkalinity [24] and its ability to effectively combat bacteria [25]. It is generally recommended to apply calcium hydroxide within the root canals and surrounding periradicular tissues, especially in cases involving sizable and chronic periapical lesions producing 80.8% success rate. This substance is believed to exert a direct influence on inflamed tissues and epithelial cystic linings, ultimately promoting periapical healing and facilitating bone repair [26]. In our case, calcium hydroxide was introduced into the root canal for a duration of three weeks, resulting in the alleviation of symptoms. As shown in Figure 9, in this case, we have employed a novel technique utilizing the synergistic effect of i-PRF combined with MTA.

An innovative formulation of Platelet-Rich Fibrin (PRF), referred to as "injectable" PRF or i-PRF, has been introduced with the aim of simplifying the application of concentrated platelets in a liquid form. This formulation can be used either independently or in combination with bioactive materials, offering promising prospects for the enhancement of both soft and hard tissue healing. It achieves this through the creation of a three-dimensional fibrin network that is abundant in platelets, type I collagen, leukocytes, growth factors, and osteocalcin [27].

i-PRF is prepared by employing a low-speed centrifugation concept, which significantly enhances the concentration of regenerative cells, leukocytes, platelets, and growth factors when compared to other PRF formulations that use higher centrifugation speeds [28]. Additionally, i-PRF plays a pivotal role in tissue healing by activating the host defense system due to its high platelet and leukocyte content. This high leukocyte count contributes to its antimicrobial potency against bacterial lipopolysaccharides when compared to other platelet concentrates. Furthermore, leukocytes are instrumental in wound healing by directing and recruiting various cell types [29]. Importantly, i-PRF contains a unique combination of proteins with diverse biochemical signals, which may work synergistically to exert a positive biological impact on tissue regeneration and mineralization. As a result, i-PRF is a promising option for tissue engineering, harnessing autologous cues to promote the regeneration of hard tissues [30].

The benefits of combining i-PRF with bioactive materials (such as MTA) include serving as a scaffold material with appropriate mechanical strength, hydrophilicity, and excellent osteoconductivity. It aids in wound healing, bone growth, maturation, material stabilization, and hemostasis, as well as improving the handling properties of bioactive materials [31]. Therefore, the combination of i-PRF and MTA for non-surgical healing of periapical lesion was used.

## Conclusions

This case report demonstrates the successful non-surgical management of a large periapical lesion through a novel combination of i-PRF and MTA. This innovative approach offers an effective alternative to traditional surgical interventions, emphasizing patient comfort and optimal tissue healing. By harnessing the regenerative potential of i-PRF and the osteoconductivity of MTA, this technique showcases promising prospects for periapical lesion treatment. Further research and clinical validation will be essential to establish its broader applicability in endodontic practice.

## References

[1] Soares J, Santos S, Silveira F, Nunes E (2006). Nonsurgical treatment of extensive cyst-like periapical lesion of endodontic origin. Int Endod J.

[2] Nair PN (1998). New perspectives on radicular cysts: do they heal?. Int Endod J.

[3] Lin LM, Ricucci D, Lin J, Rosenberg PA (2009). Nonsurgical root canal therapy of large cyst-like inflammatory periapical lesions and inflammatory apical cysts. J Endod.

[4] Salamat K, Rezai RF (1986). Nonsurgical treatment of extraoral lesions caused by necrotic nonvital tooth. Oral Surg Oral Med Oral Pathol.

[5] Kunhappan S, Kunhappan N, Saraf KK, Kridutt V (2017). Nonsurgical endodontic treatment of teeth associated with large periapical lesion using triple antibiotic paste and mineral trioxide aggregate apical plug: a case series. J Conserv Dent.

[6] Lin LM, Huang GT, Rosenberg PA (2007). Proliferation of epithelial cell rests, formation of apical cysts, and regression of apical cysts after periapical wound healing. J Endod.

[7] Moshari A, Vatanpour M, EsnaAshari E, Zakershahrak M, Jalali Ara A (2017). Nonsurgical management of an extensive endodontic periapical lesion: a case report. Iran Endod J.

[8] Walker TL, Davis MS (1984). Treatment of large periapical lesions using cannalization through involved teeth. J Endod.

[9] Calişkan MK, Sen BH (1996). Endodontic treatment of teeth with apical periodontitis using calcium hydroxide: a long-term study. Endod Dent Traumatol.

[10] Murphy WK, Kaugars GE, Collett WK, Dodds RN (1991). Healing of periapical radiolucencies after nonsurgical endodontic therapy. Oral Surg Oral Med Oral Pathol.

[11] White S, Pharoah M (2014). Oral radiology: principles and interpretation. https://books.google.co.in/books/about/Oral_Radiology_E_Book.html?id=V7PwAwAAQBAJ&redir_esc=y.

[12] Bhaskar SN (1972). Nonsurgical resolution of radicular cysts. Oral Surg Oral Med Oral Pathol.

[13] Miron RJ, Fujioka-Kobayashi M, Hernandez M, Kandalam U, Zhang Y, Ghanaati S, Choukroun J (2017). Injectable platelet rich fibrin (i-PRF): opportunities in regenerative dentistry?. Clin Oral Investig.

[14] Mansour NK, Fayyad DM, Farghaly AM (2023). Reparative calcified barrier characterization after mixing injectable-platelet rich fibrin with bioactive direct pulp capping agents; an exp. study. Study Insights Herb Med.

[15] Lalonde ER, Luebke RG (1986). The frequency and distribution of periapical cysts and granulomas. an evaluation of 800 specimens. Oral Surg Oral Med Oral Pathol.

[16] Nair PNR, Pajarola G, Schroeder HE (1996). Types and incidence of human periapical lesions obtained with extracted teeth. Oral Surg Oral Med Oral Pathol Oral Radiol Endod.

[17] Schulz M, von Arx T, Altermatt HJ, Bosshardt D (2009). Histology of periapical lesions obtained during apical surgery. J Endod.

[18] Natkin E, Oswald RJ, Carnes LI (1984). The relationship of lesion size to diagnosis, incidence, and treatment of periapical cysts and granulomas. Oral Surg Oral Med Oral Pathol.

[19] Eversole LR (1984). Clinical outline of oral pathology: diagnosis and treatment. https://catalogue.nla.gov.au/catalog/841037.

[20] Lalonde ER (1970). A new rationale for the management of periapical granulomas and cysts: an evaluation of histopathological and radiographic findings. J Am Dent Assoc.

[21] Morse DR, Patnik JW, Schacterle GR (1973). Electrophoretic differentiation of radicular cysts and granulomas. Oral Surg Oral Med Oral Pathol.

[22] Pitcher B, Alaqla A, Noujeim M, Wealleans JA, Kotsakis G, Chrepa V (2017). Binary decision trees for preoperative periapical cyst screening using cone-beam computed tomography. J Endod.

[23] Thomas K, T PD, Simon EP (2012). Management of large periapical cystic lesion by aspiration and nonsurgical endodontic therapy using calcium hydroxide paste. J Contemp Dent Pract.

[24] Abbott PV (2002). The periapical space--a dynamic interface. Aust Endod J.

[25] Sjögren U, Figdor D, Spångberg L, Sundqvist G (1991). The antimicrobial effect of calcium hydroxide as a short-term intracanal dressing. Int Endod J.

[26] Javidi M, Zarei M, Afkhami F, Majdi LM (2013). An in vitro evaluation of environmental pH changes after root canal therapy with three different types of calcium hydroxide. Eur J Dent.

[27] Choukroun J, Diss A, Simonpieri A (2006). Platelet-rich fibrin (PRF): a second-generation platelet concentrate. Part IV: clinical effects on tissue healing. Oral Surg Oral Med Oral Pathol Oral Radiol Endod.

[28] Bakhtiar H, Esmaeili S, Fakhr Tabatabayi S, Ellini MR, Nekoofar MH, Dummer PM (2017). Second-generation platelet concentrate (platelet-rich fibrin) as a scaffold in regenerative endodontics: a case series. J Endod.

[29] Karde PA, Sethi KS, Mahale SA, Khedkar SU, Patil AG, Joshi CP (2017). Comparative evaluation of platelet count and antimicrobial efficacy of injectable platelet-rich fibrin with other platelet concentrates: an in vitro study. J Indian Soc Periodontol.

[30] Fernández-Medina T, Vaquette C, Ivanovski S (2019). Systematic comparison of the effect of four clinical-grade platelet rich hemoderivatives on osteoblast behaviour. Int J Mol Sci.

[31] Mansour NK, Sharraan M, Fayad D, Abdullah Hashem M (2021). Effect of injectable-platelet rich fibrin on marginal adaptation of bioactive materials used as direct pulp capping; an experimental animal study. Dent Sci Updat.

